# Evolution of multipartite mitochondrial genomes in the booklice of the genus *Liposcelis* (Psocoptera)

**DOI:** 10.1186/1471-2164-15-861

**Published:** 2014-10-05

**Authors:** Shi-Chun Chen, Dan-Dan Wei, Renfu Shao, Jun-Xia Shi, Wei Dou, Jin-Jun Wang

**Affiliations:** Key Laboratory of Entomology and Pest Control Engineering, College of Plant Protection, Southwest University, Chongqing, P. R. China; Gene Cology Research Centre, Faculty of Science, Education and Engineering, University of the Sunshine Coast, Maroochydore, Queensland, Australia

**Keywords:** Mitochondrial genome, *Liposcelis entomophila*, *Liposcelis paeta*, Pseudogene, Evolution

## Abstract

**Background:**

The genus *Liposcelis* (Psocoptera: Troctomorpha) has more than 120 species with a worldwide distribution and they pose a risk for global food security. The organization of mitochondrial (mt) genomes varies between the two species of booklice investigated in the genus *Liposcelis. Liposcelis decolor* has its mt genes on a single chromosome, like most other insects; *L. bostrychophila*, however, has a multipartite mt genome with genes on two chromosomes.

**Results:**

To understand how multipartite mt genome organization evolved in the genus *Liposcelis*, we sequenced the mt genomes of *L. entomophila* and *L. paeta* in this study. We found that these two species of booklice also have multipartite mt genomes, like *L. bostrychophila*, with the mt genes we identified on two chromosomes. Numerous pseudo mt genes and non-coding regions were found in the mt genomes of these two booklice, and account for 30% and 10% respectively of the entire length we sequenced. In *L. bostrychophila*, the mt genes are distributed approximately equally between the two chromosomes. In *L. entomophila* and *L. paeta*, however, one mt chromosome has most of the genes we identified whereas the other chromosome has largely pseudogenes and non-coding regions. *L. entomophila* and *L. paeta* differ substantially from each other and from *L. bostrychophila* in gene content and gene arrangement in their mt chromosomes.

**Conclusions:**

Our results indicate unusually fast evolution in mt genome organization in the booklice of the genus *Liposcelis*, and reveal different patterns of mt genome fragmentation among *L. bostrychophila*, *L. entomophila* and *L. paeta*.

**Electronic supplementary material:**

The online version of this article (doi:10.1186/1471-2164-15-861) contains supplementary material, which is available to authorized users.

## Background

Animal mitochondrial (mt) genomes are typically a circular DNA molecule, 13–20 kb in size, consisting of a control region (CR) and 37 genes: 13 protein-coding genes (PCG), 2 ribosomal RNA genes (rRNA), and 22 transfer RNA genes (tRNA) [1–5]. The organization, gene content and gene arrangement of animal mt genomes are usually very conserved [4]. For insects, the ancestral mt genome organization is retained in most species known, although minor changes in gene arrangement were observed in several groups of insects [1, 6–9].

The order Psocoptera (booklice and barklice) contains more than 4,400 described species in three suborders: Troctomorpha, Trogiomorpha, and Psocomorpha [10, 11]. Complete or near complete mt genomes have been reported for three barklice (suborders Trogiomorpha and Psocomorpha) and two booklice (suborder Troctomorpha) [12–15]. The mt genomes of the three barklice, Lepidopsocidae sp. RS-2001 (suborder Trogiomorpha), *Psococerastis albimaculata* and *Longivalvus hyalospilus* (suborder Psocomorpha), retained largely the ancestral mt genome organization of insects with rearrangement of several genes in each species [12, 14]. The mt genomes of the two booklice, *Liposcelis decolor* and *L. bostrychophila* (suborder Troctomorpha), however, are highly rearranged; only one ancestral gene arrangement, *atp8-atp6*, is retained. These two booklice differ even from each other in the organization of their mt genomes. *L. decolor*, like most other insects, has the typical single-chromosome mt genome of animals [15]. *L. bostrychophila*, however, has a multipartite mt genome with two chromosomes [13].

The genus *Liposcelis* has more than 120 species with a worldwide distribution [16–19]; many of them are important pests to stored grain products [20]. *Liposcelis* species are divided into four groups phylogenetically: A, B, C and D [18, 19, 21]. *L. decolor* is in the group B whereas *L. bostrychophila* is in the group D [18]. Substantial variation in morphology and physiology among *Liposcelis* groups has been reported previously [17, 21, 22]. To understand how multipartite mt genomes evolved in the genus *Liposcelis*, we further sequenced the mt genomes of *L. entomophila* (from group A) and *L. paeta* (from group D). We found that these two booklice also have multipartite mt genomes, like *L. bostrychophila*. Further, *L. entomophila* and *L. paeta* differ substantially from each other and from *L. bostrychophila* in gene content and gene arrangement in their mt genomes. Our results indicate an unusually fast evolution in mt genome organization in the booklice of the genus *Liposcelis*.

## Methods

### Ethics statement

No specific permits were required for the insects collected in this study. The sampling locations were not privately owned or protected in any way and the collection did not involve endangered or protected species.

### Sample collection, DNA extraction and mt genome amplification

The booklice were collected at grain storage facilities. *L. entomophila* were collected in Beibei, Chongqing and *L. paeta* in Wuzhou, Guangxi, China. They were identified to species by morphology [17, 23], and partial sequences of *rrnL* and *cox1* genes [24]. Total genomic DNA was extracted from ~300 booklice specimens (20 mg) using a Tissue/Cell gDNA Mini Kit (Watson Biotechnologies, Shanghai, China) and stored at -20°C.

Partial sequences of *cox1, cob, rrnS,* and *rrnL* genes of *L. entomophila* were amplified initially by PCR with conserved insect primers [25]. Two pairs of primers, E1 - E2 and E3 - E4, were designed from *cox1* and *cob* genes (Additional file 1). Two overlapping fragments were amplified by long PCR with E1 - E2 and E3 - E4, sequenced and assembled into a contig (*L. entomophila* chromosome I) with SeqMan (DNAStar). The non-coding sequence, which contains the sites for genome replication and the initiation of gene transcription, is always shared by all of mini-chromosomes of a fragment mt genome [13, 26, 27]. Thus, a primer (E6) has been designed from a non-coding region (*NCRI-1*) in *L. entomophila* chromosome I. Another primer (E5) was designed from *rrnL* gene. With E5 - E6, a 10,231 bp long fragment was amplified and sequenced. A pair of outbound primers (E7 and E8) was designed from this fragment, and a complementary sequence of E5 - E6 was amplified. These two fragments were assembled into a contig. For *L. paeta*, fragments of *nad5*, *rrnS* and *rrnL* genes were amplified initially; four pairs of primers (P1-P2, P3-P4, P5-P6 and P7-P8) were designed from these three gene fragments. Then, four fragments were amplified with these four pairs of primers, and were assembled into two contigs (*L. paeta* chromosome I and II). To verify *L. paeta* chromosome II and avoid the mistake might be caused by primers (P6 and P8) at pseudogene *PrrnL-2*, a 3,412 bp fragment was amplified additionally with primers P9-P10 (Additional file 2).

Each long PCR reaction is 25 μL in volume, containing 1.0 μL each of forward primer (10 μM) and reverse primer (10 μM), 4.0 μL of dNTPs mix (each 2.5 mM), 1.0 μL of template DNA, 2.5 μL MgCl_2_ (25 mM), 2.5 μL of 10 × LA PCR reaction buffer II, 12.75 μL ddH_2_O and 0.25 μL LA Taq DNA polymerase (5 U/μL, Takara). All reactions were carried out using C1000^TM^ thermal cyclers (Bio-RAD, Hercules, CA, USA) with the follow conditions: 2 min denaturation at 94°C, 37 cycles of 94°C for 20 s, 58°C for 50 s, 68°C for 5–10 min (depending on target size, 1 min/kb), followed by a final extension at 68°C for 15 min. Gel-purified amplification products < 5 kb in size were ligated into pGEM-T Easy vectors (Promega, Madison, WI, USA), and introduced into *Escherichia coli* (Trans5*α*, Beijing TransGen Biotech, Beijing, China). Followed by ampicillin selection, plasmid DNAs from positive clones were sequenced with M13 primers. Longer PCR products (>5 kb) were directly sequenced with both forward and reverse PCR primers and internal primers by primer walking. All products were sequenced by Life Technologies in Guangzhou, China.

### Sequence annotation and analysis

The protein-coding genes (PCGs) were identified by the ORF Finder (http://www.ncbi.nlm.nih.gov/gorf/gorf.html) and rRNA genes by BLAST searches, then confirmed by alignment with homologous genes from those of other booklouse and louse species (Additional file 3). The transfer RNA genes were identified by cloverleaf secondary structure using ARWEN [28] with default parameters and tRNAscan-SE 1.21 [29] with the parameters: Search Mode = “EufindtRNA-Cove”, Genetic Code = “Invertebrate Mito” and Cove score cutoff = 0.1. We used Mfold Server [30] in RNA folding form with default parameters to construct the typical stem-loop secondary structure of putative control region. The base composition was analyzed with BioEdit (http://bioedit.software.informer.com/). Sequences of mt genomes of other lice and booklice were retrieved from GenBank and MitoZoa [31] (Additional file 3).

### Phylogenetic analyses

We conducted phylogenetic analyses of the mt genome sequences of seven species of Psocoptera and thirteen species of Phthiraptera. The mt genome sequence of *Drosophila melanogaster* was used as an outgroup. Sequences of all protein-coding genes and rRNA genes except *atp8* and *nad4L* were used in phylogenetic analyses. *atp8*, *nad4L* and tRNA genes were excluded because they are too short to align among the psocodean species. Two alignments were used for phylogenetic analyses: 1) a concatenated nucleotide sequence alignment of protein-coding genes and two rRNA genes (Additional file 4); 2) a concatenated amino acid sequence alignment of eleven protein-coding genes (Additional file 5). Nucleotide sequences of all protein-coding genes were aligned at the amino acid level using the default settings in ClustalW as implemented in MEGA 5 [32]; the alignments were then back-translated into the corresponding nucleotide sequences. Amino acid sequences of PCGs were aligned in ClustalW; All of the alignments were then imported into the Gblocks server (http://molevol.cmima.csic.es/castresana/Gblocks_server.html) to remove poorly aligned sites [33]. Gblocks server was applied with the ‘codons’, ‘DNA’ and ‘protein’ mode respectively for PCG nucleotide sequences, rRNA sequences and PCG amino acid sequences, and with all options for a stringent selection were chosen. Substitution saturations of the nucleotide sequences were examined using DAMBE 5.3.9 [34]. Whole PCG nucleotide sequences were selected to enter the next step if *I*ss (index of substitution saturation) was significantly lower than *I*ss.c (critical value for symmetrical tree topology) (*P* < 0.05). All of the protein-coding genes, except *nad2* and *nad3*, passed this test; the third codon positions of *nad2* and *nad3* were thus excluded from our phylogenetic analyses. The best fit models for the alignment of nucleotide sequence and amino acid sequence were determined using the Akaike Information Criterion in jModelTest 2.1.4 [35, 36] and ProtTest 3.2 [37], then the GTR + I + G model and MtArt + I + G + F model were chosen. Phylogenetic trees were constructed from the dataset using maximum likelihood (ML) method. ML analyses were performed using PhyML3.0 (http://www.atgc-montpellier.fr/phyml/) [38] with substitution model ‘GTR’ or ‘MtArt’, type of tree improvement “SPR & NNI”, and the shape parameter and the propotion of invariable sites was estimated by jModelTest 2.1.4 and ProtTest 3.2.

## Results

### Mitochondrial genomes of *L. entomophila*and *L. paeta*

**Figure 1:**
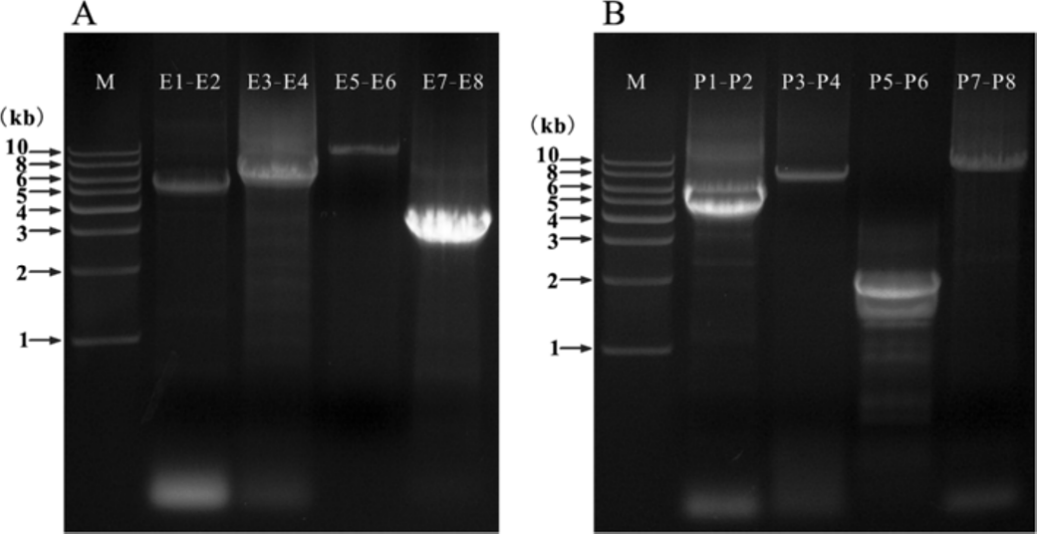
**PCR amplicons from mitochondrial DNA of** ***Liposcelis entomophila*** **(A) and** ***L. paeta*** **(B).** Lane M: 1 kb marker (Biomed). “E1-E2”, the product of PCR with primers E1 and E2, etc. Details of primers are in Additional files 1 and 2.

**Figure 2:**
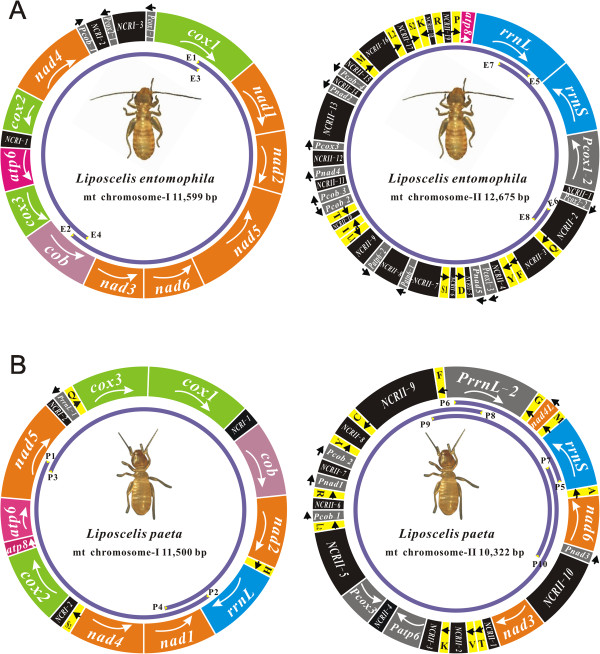
**The mitochondrial genomes of** ***Liposcelis entomophila*** **(A) and** ***L. paeta*** **(B).** The transcriptional orientation is indicated with arrows. Protein-coding genes, ribosomal RNA genes and transfer RNA genes are shown in bright colors. All of them in the map follow standard abbreviations. tRNA genes for the two serine and two leucine tRNAs: S_1_ = AGN, S_2_ = UCN, L_1_ = CUN and L_2_ = UUR. Pseudogenes are shown in gray. The non-coding sequences >100 bp are indicated in black. Arrows and purple curves indicate primers and PCR fragments, respectively. See Additional files 1 and 2 for PCR primers.

The mt genome of *L. entomophila* consists of two circular chromosomes, I (GenBank Accession No. KF649223) and II (GenBank Accession No. KF649224). Chromosome I, 11,599 bp long, was assembled from two overlapping PCR amplicons, 5,634 bp (E1 - E2) and 6,413 bp (E3 - E4), respectively (Figures 1A and 2A). These two amplicons overlap by 64 bp in *cox1* and 284 bp in *cob*. The other two amplicons, E5 - E6 (10,231 bp) and E7 - E8 (3,353 bp), were assembled to form chromosome II, 12,675 bp long; these two amplicons overlap by 347 bp in *rrnL* and 470 bp in *NCRII-3* (Figures 1A and 2A). Totally, 28 of the 37 mt genes typical of bilateral animals and 15 pseudogenes were found in the two mt chromosomes of *L. entomophila*. Chromosome I contains 11 protein-coding genes (*atp6*, *cob*, *cox1*-*cox3*, and *nad1*-*nad6*) and three pseudogenes. Chromosome II contains two rRNA genes (*rrnL* and *rrnS*), 14 tRNA genes (*trnD*, *trnF*, *trnI*, *trnK*, *trnL1*, *trnL2*, *trnM*, *trnP*, *trnQ*, *trnR*, *trnS1*, *trnS2*, *trnT* and *trnY*) and a PCG (*atp8*) (Figure 2A and Additional file 6). Additionally, 12 pseudogenes were also found on this chromosome (Figure 2A and Additional file 7).

The mt genome of *L. paeta* also had two chromosomes: I (GenBank Accession No. KF649226) and II (GenBank Accession No. KF649225). These two chromosomes were 11,500 bp and 10,322 bp long. Chromosome I was assembled from two overlapping fragments, P1 - P2 (4,885 bp) and P3 - P4 (7,659 bp). Chromosome II was assembled from P5 - P6 (1,860 bp) and P7 - P8 (8,908 bp). P1 - P2 and P3 - P4 overlap by 218 bp in *nad5* and 731 bp in *rrnL*, and the other two fragments overlap by 117 bp in *rrnS* and 233 bp in *PrrnL-2* (Figures 1B and 2B). To verify these contigs, a 3,412 bp fragment (P9 - P10) was amplified additionally with primers P9 - P10 (Additional file 2). We found 29 of the 37 mt genes typical of bilateral animals and 8 pseudogenes in *L. paeta*. Chromosome I contains 10 protein-coding genes (*atp6*, *atp8*, *cob*, *cox1*-*cox3*, *nad1*, *nad2*, *nad4*, and *nad5*), an rRNA gene (*rrnL*), three tRNA genes (*trnS2*, *trnQ*, and *trnH*) and a pseudogene. Chromosome II contains three protein-coding genes (*nad3*, *nad4L*, and *nad6*), *rrnS*, 11 tRNA genes (*trnA*, *trnC*, *trnF*, *trnG*, *trnK*, *trnL2*, *trnM*, *trnR*, *trnT*, *trnV* and *trnY*) and seven pseudogenes (Figures 2B, Additional files 6 and 8).

**Figure 3:**
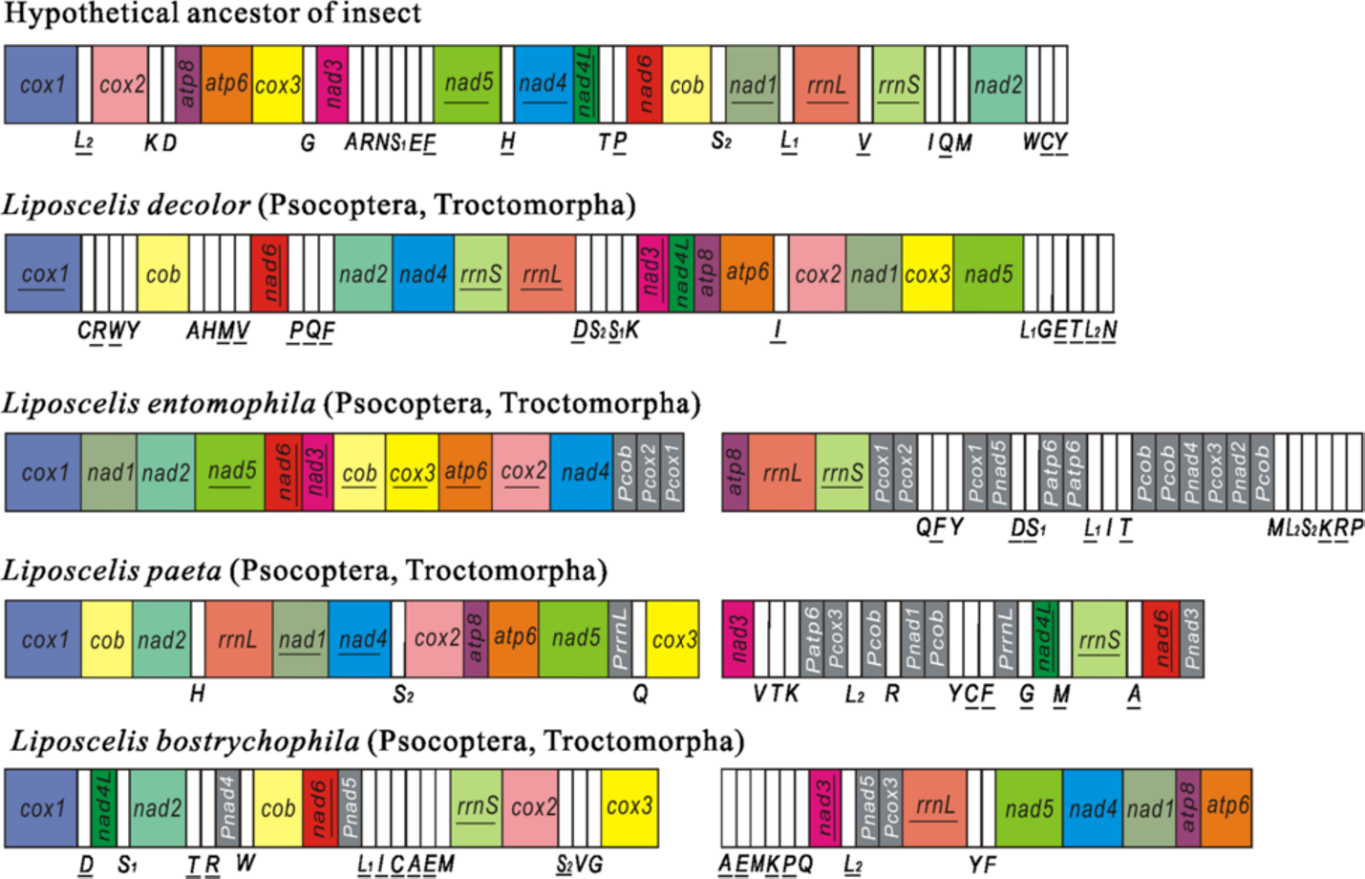
**Arrangement of mitochondrial genes in** ***Liposcelis*** **and the hypothetical ancestor of the insect.** Circular genomes have been arbitrarily linearized for ease of comparison. Gene names are the standard abbreviations used in the present study. tRNA genes are designated by the single letter according to the IPUC-IUB one-letter amino acid codes. Genes which are underlined are encoded on the opposite strand to the majority of genes in that particular genome. Gray and white boxes represent pseudogenes and transfer RNA genes, respectively. The boxes in bright colors represent 13 protein-coding genes and 2 ribosomal RNA genes.

The mt gene arrangements of *L. entomophila* and *L. paeta* differ from that of the hypothetical ancestor of insects, from that of the other booklice and from each other. *L. entomophila* shares no mt gene boundary with the hypothetical ancestor of insects, *L. paeta*, *L. bostrychophila*, and *L. decolor*. For *L. paeta*, only *atp8*-*atp6* is shared with the hypothetical ancestor of insects, *L. bostrychophila* and *L. decolor*, and *cox3-cox1* and *nad4*-*nad1* are shared with *L. bostrychophila*, which is also from the group D of *Liposcelis* species (Figure 3).

### Pseudogenes and non-coding sequences

We identified 15 and 8 pseudo mt genes in *L. entomophila* and *L. paeta*, respectively (Table 1). The total length of all of the 15 pseudogenes in *L. entomophila* is 2,555 bp, which accounts for 10.53% of the total length of the two chromosomes. For *L. paeta*, the total length of all of the 8 pseudogenes is 2,466 bp, which accounts for 11.30% of the two mt chromosomes we sequenced. Each of the mt pseudogenes is a partial sequence of a protein-coding gene or an rRNA gene with several nucleotides changed, and has a high similarity (>85%) to the homologous sequence in functional gene (Table 1). For instance, *Pcox1-2* of *L. entomophila*, located on the chromosome II, is 847 bp long and is identical to the part of *cox1* from 163 to 1,009 bp except for a single nucleotide change (Additional file 9).

**Table 1:** **Pseudogenes in the mitochondrial genomes of** ***Liposcelis entomophila*** **and** ***L. paeta***

Species	Pseudogene	Size	Counterpart in full-length gene	Identity (%)
*L. entomophila*	*Patp6-1*	49	454-502	100.00
*L. entomophila*	*Patp6-2*	98	543-642	98.00
*L. entomophila*	*Pcob-1*	44	1025-1068	90.91
*L. entomophila*	*Pcob-2*	186	212-399	97.87
*L. entomophila*	*Pcob-3*	207	834-1048	86.76
*L. entomophila*	*Pcob-4*	119	366-484	99.16
*L. entomophila*	*Pcox1-1*	122	1005-1130	95.24
*L. entomophila*	*Pcox1-2*	847	163-1009	99.88
*L. entomophila*	*Pcox1-3*	191	270-460	99.48
*L. entomophila*	*Pcox2-1*	124	290-413	100.00
*L. entomophila*	*Pcox2-2*	129	530-660	97.71
*L. entomophila*	*Pcox3*	74	18-91	98.65
*L. entomophila*	*Pnad2*	108	650-757	90.74
*L. entomophila*	*Pnad4*	125	689-810	89.60
*L. entomophila*	*Pnad5*	132	297-429	99.25
*L. paeta*	*Patp6*	562	96-657	99.82
*L. paeta*	*Pcob-1*	62	772-833	100.00
*L. paeta*	*Pcob-2*	92	870-961	98.91
*L. paeta*	*Pcox3*	422	237-660	98.82
*L. paeta*	*Pnad1*	62	680-741	98.39
*L. paeta*	*Pnad3*	164	144-315	95.35
*L. paeta*	*PrrnL-1*	188	836-1025	94.24
*L. paeta*	*PrrnL-2*	914	111-1025	99.02

**Figure 4:**
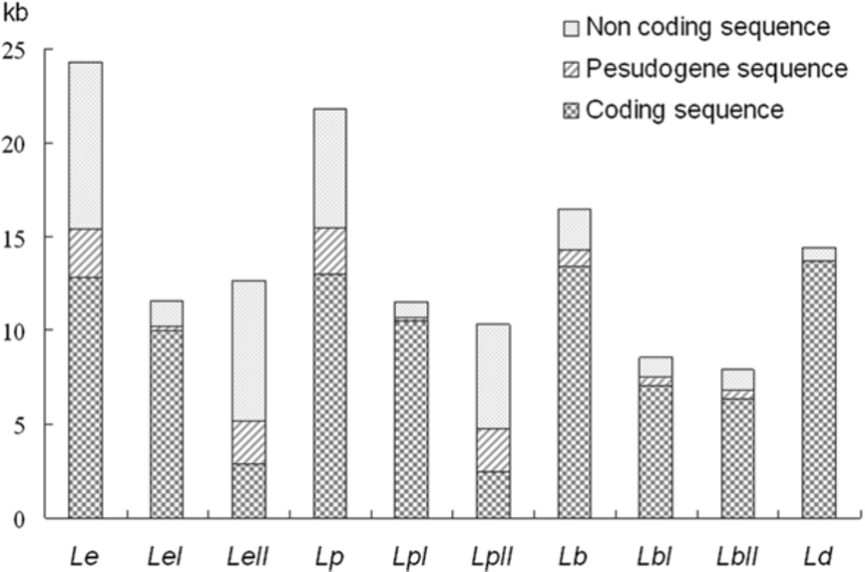
**The proportion of coding sequence, pseudogene and non-coding sequence in booklice mitochondrial genomes.** Species are abbreviated as following: *Le*, *Liposcelis entomophila*; *LeI*, *Liposcelis entomophila* mt chromosome I; *LeII*, *Liposcelis entomophila* mt chromosome II; *Lp*, *Liposcelis paeta*; *LpI*, *Liposcelis paeta* mt chromosome I; *LpII*, *Liposcelis paeta* mt chromosome II; *Lb*, *Liposcelis bostrychophila*; *LbI*, *Liposcelis bostrychophila* mt chromosome I; *LbII*, *Liposcelis bostrychophila* mt chromosome II; *Ld*, *Liposcelis decolor*.

**Figure 5:**
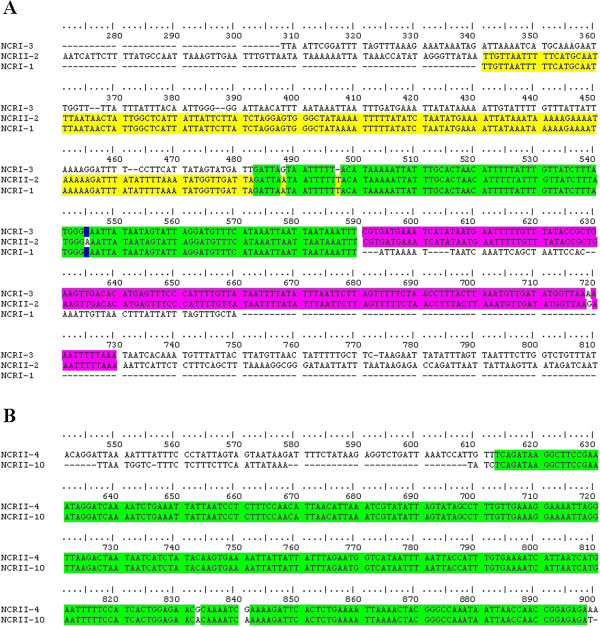
**Alignment of nucleotide sequences in the non-coding regions of the mitochondrial genomes of** ***Liposcelis entomophila*** **(A) and** ***Liposcelis paeta*** **(B).** The green highlight shows the concensus sequence of the three non-coding regions in *Liposcelis entomophila* **(A)** and the two in *L. paeta* **(B)**, yellow shows the concensus sequence of *NCRI-1* and *NCRII-2*, purple shows that of *NCRI-3* and *NCRII-2* and blue shows that of *NCRI-1* and *NCRI-3* in *L. entomophila* **(A)**.

Non-coding sequences also account for large proportions of the mt chromosomes of *L. entomophila* and *L. paeta*. The non-coding sequences are 8,912 bp and 6,391 bp long, in total, for *L. entomophila* and *L. paeta*, and account for 36.72% and 29.29% of the entire length of their mt chromosomes. There are 22 and 13 non-coding sequences that are longer than 100 bp in the mt chromosomes of *L. entomophila* and *L. paeta*, respectively. For both *L. entomophila* and *L. paeta*, pseudogenes and non-coding sequences are largely on one of the mt chromosomes (chromosome II), whereas coding sequences are on the other chromosome (chromosome I) (Figures 2 and 4). Intriguingly, partial sequences are shared by three or two non-coding regions in *L. entomophila* and *L. paeta*. Three non-coding regions (*NCRI-1*, *NCRI-3* and *NCRII-2*) of *L. entomophila*, each two of the three share a consistent sequence, and a partial sequence is common in all of the three. In *L. paeta*, two non-coding regions from mt chromosome II (*NCRII-5* and *NCRII-10*) contain the same 286 bp sequence with two nucleotides change (Figure 5).

### Phylogenetic relationships among species of Psocodea

**Figure 6:**
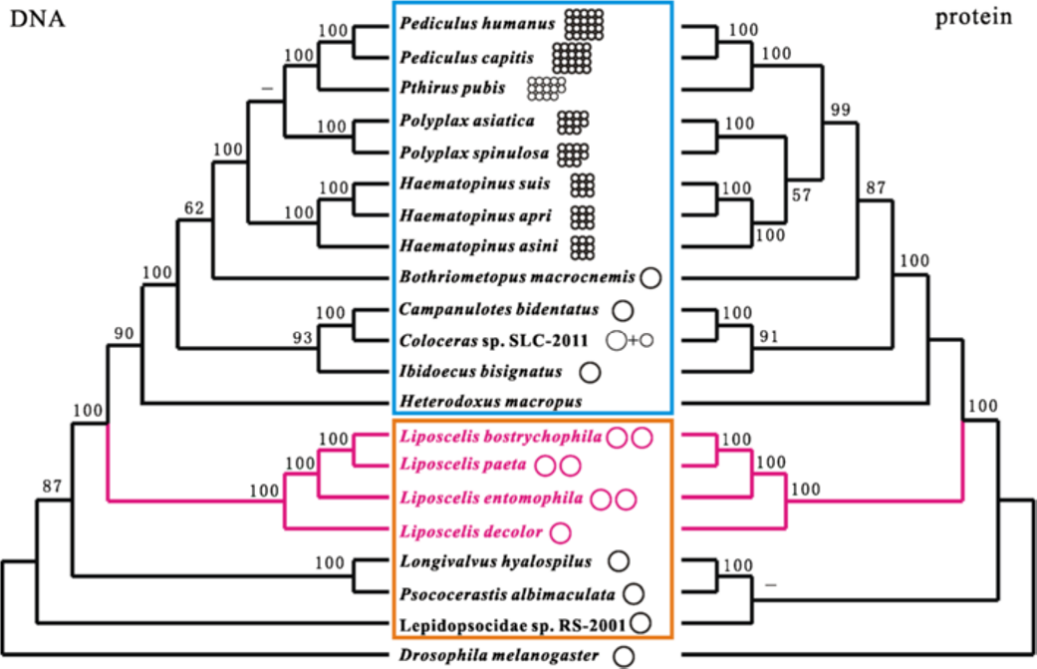
**Phylogeny from Psocodea mitochondrial genome sequences.** Numbers above the left branches show ML bootstrap support values for the phylogenies from nucleotide sequences, the right from amino acid sequences. Only support above 50% is shown. The insects belong to Phthiraptera and Psocoptera are shown in blue and orange frames, respectively. The circles besides names are a schematic representation of the mitochondrial genome organization.

We analyzed the mt genome sequences with maximum likelihood (ML) method to infer the phylogenetic relationship of *L. entomophila*, *L. paeta*, *L. bostrychophila*, *L. decolor* and with other species of Psocodea (Figure 6). In the phylogenetic tree, the four *Liposcelis* species were clustered together with strong support (100% bootstrap values). Within the genus *Liposcelis*, *L. paeta* formed a clade with *L. bostrychophila*; these two species, which were from group D [18], were most closely related to *L. entomophila* (group A).

## Discussion

### Variation in the pattern of multipartite mt genome evolution among the three *Liposcelis*species

While *L. decolor* has the typical one-chromosome mt genome, like most other insects and animals. *L. entomophila*, *L. paeta* and *L. bostrychophila* have multipartite mt genomes. For these three *Liposcelis* species, there are extensive variations in the pattern of multipartite mt genome evolution. Firstly, the presence of numerous pseudogenes and non-coding sequences in *L. entomophila* and *L. paeta* makes their mt genomes much larger than that of *L. bostrychophila* (16,463 bp) (Figure 4). Furthermore, the three *Liposcelis* species that have multipartite mt genomes differ from each other in the distribution of mt genes, pseudogenes, and non-coding sequences between the mt chromosomes. The two mt chromosomes of *L. bostrychophila* contain nearly equal amounts of genes, pseudogenes and non-coding sequences. For *L. entomophila* and *L. paeta*, however, most of the mt genes are on one chromosome, whereas pseudogenes and non-coding sequences are on the other chromosome (Figures 2 and 4). For numerous non-coding sequences, the two mt chromosomes of *L. bostrychophila* contain the same major non-coding region (*NCRI-4* and *NCRII-3*) [13]. However, three non-coding regions from two chromosomes of *L. entomophila* share partial sequences and two non-coding regions from mt chromosome II of *L. paeta* share a 286 bp sequence (Figure 5). For fragmented mt genome of blood-sucking lice [26, 27, 39–41] and the rotifers [42, 43], mini-chromosomes usually have similar major non-coding sequences. The arrangement of mt genes varies extensively among the three *Liposcelis* species; indeed, no gene boundary is shared by the three booklice. The two group-D species, *L. paeta* and *L. bostrychophila*, share three gene boundaries, *atp8-atp6*, *cox3-cox1* and *nad4*-*nad1* (Figure 3). Prior to this study, intra-genus variations of mt gene arrangement have been reported in several animal genera, such as *Haematopinus* [39], *Polyplax* [40], *Brachionus* [42], *Ciona* [44], *Phallusia* [45], *Corallium* [46], *Schistosoma* [47], *Leptotrombidium* [48, 49], and *Dermatophagoides* [50]. However, the extent of variation in these genera is much lower than that in the genus *Liposcelis*.

### Pseudo mt genes and duplicated non-coding sequences in the *Liposcelis*species

**Figure 7:**
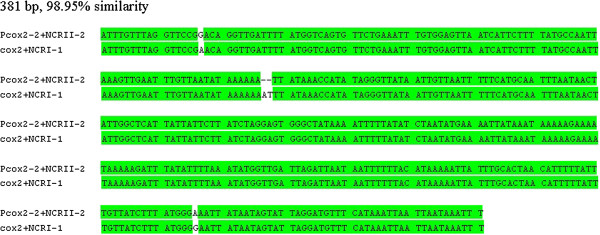
**Alignment of nucleotide sequences of “** ***cox2*** **+** ***NCRI-1*** **” from chromosome I and “** ***Pcox2-2*** **+** ***NCRII-2*** **” in** ***Liposcelis entomophila*** **.** Consensus sequences are shown in the green background.

Prior to the present study, pseudo mt genes have been described in *L. bostrychophila* and numerous other animals. Most of the pseudo mt genes are short and derived from tRNAs [40, 51–56]. Pseudo mt genes longer than 100 bp, derived from protein-coding genes, were also reported [13, 57–60]. Mt gene rearrangements are usually explained by a tandem duplication-random loss (TDRL) model [61, 62], and pseudo mt genes are considered to be redundant genes in the process of being eliminated [53, 54, 57]. The large numbers of pseudo mt genes we found in *L. entomophila* and *L. paeta* in this study and in *L. bostrychophila* [13] are extraordinary for insects and animals, indicating that large-scale changes, such as tandem duplication or mt chromosome duplication, occurred relatively recently in the multipartite mt genomes of these booklice. Intriguingly, no pseudo mt gene has been found in *L. decolor*, although it also has numerous gene rearrangements like the other three *Liposcelis* species that have multipartite mt genomes [15]. It infers that mt genome fragmenting is the important inducement for pseudo mt gene appearance in booklice. For fragmented mt genomes, mt pseudogenes were found in the human pubic louse [26], the *Polyplax* rat lice [40], the *Globodera* nematodes [59, 60] and the chimeric mt minichromosomes of the human body louse [63]. The explanation for the pseudo mt gene present in these species is that a recombinatorial mechanism is responsible for their production. This explanation may be applicable for multipartite mt genome in *Liposcelis*, pseudo mt gene would be residues of interchromosome recombination. Duplicated non-coding sequence present coincided with pseudo mt gene in booklice multipartite mt genomes. The sequences, “*cox2* + *NCRI-1*” from chromosome I and “*Pcox2-2* + *NCRII-2*” from chromosome II in *L. entomophila*, have a 98.95% similarity and four nucleotides changed (Figure 7). This indicats that “*Pcox2-2* + *NCRII-2*” is generated as an entire block and derived from “*cox2* + *NCRI-1*”. Therefore, the causation of duplicated non-coding sequences present in booklice might be consistent with that of pseudo mt genes. The same situation has also been found in *L. bostrychophila* [13] and human body louse [63].

### Phylogenetic relationships among *Liposcelis*species inferred from mitochondrial genome sequences and organization

Although *L. decolor* differ from the other three *Liposcelis* species in mt genome organization, these four booklice are more closely related to each other than to other species in the Psocodea, that also echoes previous researches [16–18, 23]. In the phylogenetic tree, *L. decolor* (group B), representing the booklice with typical single chromosome mt genome, was split off from the others firstly, and the three booklice with multipartite mt genome were clustered into the same branch. This was also indicated by sequence analysis of the ribosomal internal transcribed spacers region [21]. This suggested that the multipartite mt genome organization observed in booklice likely evolved recently after *L. decolor* split from the most recent common ancestor. However, all of the blood-sucking lice (suborder Anoplura) in a branch have extreme fragmented mt genomes, that mt genome fragment began from the common ancestor of the suborder Anoplura. It can be inferred that the multipartite mt genome organization in *Liposcelis* evolved independently from fragmented mt genomes exist in the blood-sucking lice. For three booklice with multipartite mt genome, *L. paeta* (group D) and *L. bostrychophila* (group D) were clustered together, and formed a sister clade to *L. entomophila* (group A). This result is consistent with the previous studies [17, 21, 23] and can be concluded by mt gene arrangement analyses. *L. paeta* share three gene boundaries with *L. bostrychophila*, but share none of gene boundary with *L. entomophila*. However, genome size, gene distribution and A + T content show *L. entomophila* and *L. paeta* have more close relationship to each other. Mt gene rearrangement is a common phenomenon in Psocodea, and is occurred even more seriously in booklice. The gene boundaries shared by *L. paeta* and *L. bostrychophila* as powerful evidences to support the close relationship between them. Then, the disparities of genome size, gene distribution and A + T content may indicate different evolution speeds and patterns of multipartite mt genome in *Liposcelis* species.

## Conclusions

We sequenced the complete mitochondrial genome of the booklice, *L. entomophila* and *L. paeta*. Both of these two mitochondrial genomes are multipartite like *L. bostrychophila*. Numerous pseudogenes and non-coding regions have been found in these mt genomes, and one mt chromosome has most of protein-coding genes whereas the other chromosome has largely pseudogenes and non-coding regions. However, the mt genes are distributed approximately equally between the two chromosomes in *L. bostrychophila. L. entomophila* and *L. paeta* differ substantially from each other and from *L. bostrychophila* in gene content and gene arrangement in their mt chromosomes. Although the four *Liposcelis* species are different from each other in mt genome organization, they were clustered together with strong support in the phylogenetic tree. Then, these results indicate unusually fast evolution in mt genome organization in the booklice of the genus *Liposcelis*, and reveal different patterns of mt genome fragmentation among *L. bostrychophila*, *L. entomophila* and *L. paeta*.

### Availability of supporting data

The data sets supporting the results of this article are included within the article and its additional files. The nucleotide sequences of the mt genomes of the two booklice supporting the results of this article have been deposited in GenBank (accession numbers KF649223-KF649226). Phylogenetic data are available in TreeBase under study number 16391.

## Electronic supplementary material

Additional file 1: **PCR primers used for amplification of the mitochondrial genome of** ***Liposcelis entomophila*** **.** (DOC 50 KB)

Additional file 2: **PCR primers used for amplification of the mitochondrial genome of** ***Liposcelis paeta*** **.** (DOC 53 KB)

Additional file 3: **Species of insects included in phylogenetic analysis in this study.** (DOC 46 KB)

Additional file 4: **Alignments of nucleotide sequence used for phylogenetic inference.** Species are abbreviated as following: Lp, *Liposcelis paeta*; Le, *Liposcelis entomophila*; Lb, *Liposcelis bostrychophila*; Ld, *Liposcelis decolor*; Lh, *Longivalvus hyalospilus*; Pa, *Psococerastis albimaculata*; Ls, Lepidopsocidae sp. RS-2001; Bm, *Bothriometopus macrocnemis*; Cb, *Campanulotes bidentatus*; Cs: *Coloceras* sp. SLC-2011; Hm, *Heterodoxus macropus*; Ib, *Ibidoecus bisignatus*; Pc, *Pediculus capitis*; Ph, *Pediculus humanus*; Pp, *Pthirus pubis*; Hs, *Haematopinus suis*; Ha, *Haematopinus apri*; Has: *Haematopinus asini*; Poa, *Polyplax asiatica*; Pos, *Polyplax spinulosa*; Dm, *Drosophila melanogaster*. (ZIP 41 KB)

Additional file 5: **Alignments of amino acid sequence used for phylogenetic inference.** Species are abbreviated as following: Lp, *Liposcelis paeta*; Le, *Liposcelis entomophila*; Lb, *Liposcelis bostrychophila*; Ld, *Liposcelis decolor*; Lh, *Longivalvus hyalospilus*; Pa, *Psococerastis albimaculata*; Ls, Lepidopsocidae sp. RS-2001; Bm, *Bothriometopus macrocnemis*; Cb, *Campanulotes bidentatus*; Cs: *Coloceras* sp. SLC-2011; Hm, *Heterodoxus macropus*; Ib, *Ibidoecus bisignatus*; Pc, *Pediculus capitis*; Ph, *Pediculus humanus*; Pp, *Pthirus pubis*; Hs, *Haematopinus suis*; Ha, *Haematopinus apri*; Has: *Haematopinus asini*; Poa, *Polyplax asiatica*; Pos, *Polyplax spinulosa*; Dm, *Drosophila melanogaster*. (ZIP 18 KB)

Additional file 6: **Putative secondary structures of the tRNA genes identified in the mitochondrial genome of** ***Liposcelis entomophila (*** ***Le*** **) and** ***L. paeta*** **(** ***Lp*** **).** Bars indicate Watson-Crick base pairings, and dots between G and U pairs mark canonical base pairings appearing in RNA. (TIFF 721 KB)

Additional file 7: **Summary of the mitochondrial genome of** ***Liposcelis entomophila*** **.** ^a^genes and pseudogenes located in the different strand from that of *cox1* are underlined. ^b^inc = intergenic nucleotides, indicates gap nucleotides (positive value) or overlapped nucleotides (negative value) between two adjacent genes. ^c^AT-skew = (A-T)/(A + T), GC-skew = (G-C)/(G + C). ^d^genes and pseudogenes located in the different strand from that of *atp8* are underlined. (DOC 151 KB)

Additional file 8: **Summary of the mitochondrial genome of** ***Liposcelis paeta*** **.** ^a^genes and pseudogenes located in the different strand from that of *cox1* are underlined. ^b^inc = intergenic nucleotides, indicates gap nucleotides (positive value) or overlapped nucleotides (negative value) between two adjacent genes. ^c^AT-skew = (A-T)/(A + T), GC-skew = (G-C)/(G + C). ^d^genes and pseudogenes located in the different strand from that of *nad3* are underlined. (DOC 122 KB)

Additional file 9: **Alignments of putative the pseudogene** ***Pcox1-2*** **and putative functional gene** ***cox1*** **of** ***Liposcelis entomophila*** **.** Consensus sequences are shown in the green background. (TIFF 5 MB)

## Abbreviations

PCGs: Protein-coding genes
*atp6* and *atp8*: Genes for the ATPase subunits 6 and 8
*cox1*-*cox3*: Genes for cytochrome C oxidase subunits I-III
*cob*: A gene for apocytochrome b
*nad1*-*nad6* and *nad4L*: Genes for NADH dehydrogenase subunits 1–6 and 4 L
*rrnL*: Large (16S) rRNA subunit (gene)
*rrnS*: Small (12S) rRNA subunit (gene)
*trnX* (where X is replaced by one letter amino acid code of the corresponding amino acid): transfer RNA.
