# Supplementary material for: Evolution of multipartite mitochondrial genomes in the booklice of the genus Liposcelis (Psocoptera)
Source: BMC Genomics. 2014 Oct 5;15(1):861. doi: 10.1186/1471-2164-15-861 (PMC4197233; doi:10.1186/1471-2164-15-861)
Supplement: Supplementary file 1 — Additional file 1: PCR primers used for amplification of the mitochondrial genome of Liposcelis entomophila . (DOC 50 KB) [file 12864_2014_6535_MOESM1_ESM.doc]

Additional file 1. PCR primers used for amplification of the mitochondrial genome of *Liposcelis entomophila*

| Gene | Primer | Primer sequence (5’-3’) | Tm (ºC) | Amplicon size (bp) |
| --- | --- | --- | --- | --- |
| *cox1* | F91-3 | CCGGATCCTTYTGRTTYTTYGGNCAYCC | 53 | F91-3- R369-2: 376 |
| *cox1* | R369-2 | CCGGATCCACNACRTARTANGTRTCRTG |
| *cox3* | C3-J-5014 | TTATTTATTGCATCAGAAGT | 48 | C3-J-5014-C3-N-5460: 445 |
| *cox3* | C3-N-5460 | TCAACAAAGTGTCAGTATCA |
| *cob* | CBF1 | TATGTACTACCATGAGGACAAATATC | 54 | CBF1-CBR1: 433 |
| *cob* | CBR1 | ATTACACCTCCTAATTTATTAGGAAT |
| *rrnL* | 16Sar | CGCCTGTTTAACAAAAACAT | 51 | 16Sar-16Sbr: 465 |
| *rrnL* | 16Sbr | CCGGTCTGAACTCAGATCACGT |
| *rrnS* | SR-J14197 | GTACAYCTACTATGTTACGACTT | 48 | SR-J14197-SR-N14745: 535 |
| *rrnS* | SR-N14745 | GTGCCAGCAGYYGCGGTTANAC |
| *cox1* | E1 | TGCTATATTATCTATTGGTAGGTTG | 58 | E1-E2:5,634 |
| *cob* | E2 | TGATAGTAGAATGGATTTGAGGAGG |
| *cox1* | E3 | GCGATAATCATTGTAGCAGATGTGA | 58 | E3-E4: 6,413 |
| *cob* | E4 | GGCTGAATATGAAGAGGTGTAACTA |
| *rrnL* | E5 | TTTTATTATTGTGCTGTTATCCCT | 58 | E5-E6: 10,231 |
| *NCRI-1* | E6 | ATACCGCTGAAGTTGACACATGAG |
| *rrnL* | E7 | CAACATAATAACGACAGTTTAAC | 58 | E7-E8: 3,353 |
| *NCRII-2* | E8 | CCATAATGTAATAGTTGACCCTCT |
