# Supplementary material for: Evolution of multipartite mitochondrial genomes in the booklice of the genus Liposcelis (Psocoptera)
Source: BMC Genomics. 2014 Oct 5;15(1):861. doi: 10.1186/1471-2164-15-861 (PMC4197233; doi:10.1186/1471-2164-15-861)
Supplement: Supplementary file 2 — Additional file 2: PCR primers used for amplification of the mitochondrial genome of Liposcelis paeta . (DOC 53 KB) [file 12864_2014_6535_MOESM2_ESM.doc]

Additional file 2. PCR primers used for amplification of the mitochondrial genome of *Liposcelis paeta*

| Gene | Primer | Primer sequence (5’-3’) | Tm (ºC) | Amplicon size (bp) |
| --- | --- | --- | --- | --- |
| *cox1* | UEA5 | AGTTTTAGCAGGAGCAATTACTAT | 53 | UEA5-UEA8: 641 |
| *cox1* | UEA8 | AAAAATGTTGAGGGAAAAATGTTA |
| *cob* | CBF1 | TATGTACTACCATGAGGACAAATATC | 54 | CBF1-CBR1: 433 |
| *cob* | CBR1 | ATTACACCTCCTAATTTATTAGGAAT |
| *nad5* | N5-J7077 | TTAAATCCTTWGARTAAAAYCC | 48 | N5-J7077-N5-N7793: 679 |
| *nad5* | N5-N7793 | TTAGGTTGRGATGGNYTAGG |
| *rrnL* | 16Sar | CGCCTGTTTAACAAAAACAT | 51 | 16Sar-16Sbr: 438 |
| *rrnL* | 16Sbr | CCGGTCTGAACTCAGATCACGT |
| *rrnS* | SR-J14197 | GTACAYCTACTATGTTACGACTT | 48 | SR-J14197- SR-N14745: 478 |
| *rrnS* | SR-N14745 | GTGCCAGCAGYYGCGGTTANAC |
| *nad5* | P1 | CTGATGTGTCTCATAGTGGAAAAAGC | 58 | P1-P2: 4,885 |
| *rrnL* | P2 | ATGACCTCGATGTTGAATTAGG |
| *nad5* | P3 | ATTGATGGCTGCTTCCCTAACTA | 58 | P3-P4: 7,659 |
| *nad1* | P4 | TCTTTATTCGTTGGGGGTTTATGC |
| *rrnS* | P5 | CAGGAGACGACGGGCAGTTTGT | 58 | P5-P6: 1,860 |
| *rrnL* | P6 | TCTAGTTTAAGCTAAATTGAGGAATC |
| *rrnS* | P7 | GTCAGGTAAAGACGAAGATTATAG | 58 | P7-P8: 8,908 |
| *rrnL* | P8 | TAGTAAACCAAATGGCAGATTAGA |
| *NCRII-9* | P9 | TCTTGTTATTGGTGCTGTGATTAG | 58 | P9-P10: 3,412 |
| *NCRII-10* | P10 | AAAAAGGTTGAGTGTTGTTTGGCA |
