# Supplementary material for: Evolution of multipartite mitochondrial genomes in the booklice of the genus Liposcelis (Psocoptera)
Source: BMC Genomics. 2014 Oct 5;15(1):861. doi: 10.1186/1471-2164-15-861 (PMC4197233; doi:10.1186/1471-2164-15-861)
Supplement: Supplementary file 3 — Additional file 3: Species of insects included in phylogenetic analysis in this study. (DOC 46 KB) [file 12864_2014_6535_MOESM3_ESM.doc]

Additional file 3. Species of insects included in phylogenetic analysis in this study

| Species | Classification | GenBank acc. No. |
| --- | --- | --- |
| *Liposcelis entomophila* | Psocoptera: Troctomorpha | KF649223-4 |
| *Liposcelis paeta* | Psocoptera: Troctomorpha | KF649225-6 |
| *Liposcelis decolor* | Psocoptera: Troctomorpha | JX870621 |
| *Liposcelis bostrychophila* | Psocoptera: Troctomorpha | JN645275–76 |
| *Psococerastis albimaculata* | Psocoptera: Psocomorpha | JQ910989 |
| *Longivalvus hyalospilus* | Psocoptera: Psocomorpha | JQ910986 |
| Lepidopsocidae sp. RS-2001 | Psocoptera: Trogiomorpha | AF335994 |
| *Bothriometopus macrocnemis* | Phthiraptera: Ischnocera | EU183542 |
| *Campanulotes bidentatus* | Phthiraptera: Ischnocera | AY968672 |
| *Coloceras* sp. SLC-2001 | Phthiraptera: Ischnocera | JN122000 (14, 868 bp), JN122001 (7, 650 bp) |
| *Ibidoecus bisignatus* | Phthiraptera: Ischnocera | JN122005 |
| *Heterodoxus macropus* | Phthiraptera: Amblycera | AF270939 |
| *Haematopinus suis* | Phthiraptera: Anoplura | KC814602–10 |
| *Haematopinus apri* | Phthiraptera: Anoplura | KC814611–19 |
| *Haematopinus asini* | Phthiraptera: Anoplura | KF939318, KF939322, KF939324, KF939326, KJ434034-KJ434038 |
| *Pediculus humans* | Phthiraptera: Anoplura | FJ499473–90 |
| *Pediculus capitis* | Phthiraptera: Anoplura | JX080388-407 |
| *Pthirus pubis* | Phthiraptera: Anoplura | JQ976018, EU219987-95, HM241895-98 |
| *Polyplax asiatica* | Phthiraptera: Anoplura | KF647751–61 |
| *Polyplax spinulosa* | Phthiraptera: Anoplura | KF647762–72 |
| *Drosophila melanogaster* | Diptera | AF200829 |
