# Supplementary material for: Evolution of multipartite mitochondrial genomes in the booklice of the genus Liposcelis (Psocoptera)
Source: BMC Genomics. 2014 Oct 5;15(1):861. doi: 10.1186/1471-2164-15-861 (PMC4197233; doi:10.1186/1471-2164-15-861)
Supplement: Supplementary file 7 — Additional file 7: Summary of the mitochondrial genome of Liposcelis entomophila . agenes and pseudogenes located in the different strand from that of cox1 are underlined. binc = intergenic nucleotides, indicates gap nucleotides (positive value) or overlapped nucleotides (negative value) between two adjacent genes. cAT-skew = (A-T)/(A + T), GC-skew = (G-C)/(G + C). dgenes and pseudogenes located in the different strand from that of atp8 are underlined. (DOC 151 KB) [file 12864_2014_6535_MOESM7_ESM.doc]

Additional file 7. Mitochondrial genome of *Liposcelis entomophila*

A. Mitochondria chromosome I of *L. entomophila*

| Gene^a^ | Region | Size | INC^b^ | AT% | AT-skew^b^ | GC-skew^c^ | Start codon | Stop codon |
| --- | --- | --- | --- | --- | --- | --- | --- | --- |
| *cox1* | 1-1530 | 1530 | 90 | 73.59 | -0.171 | 0.054 | ATT | TAA |
| *nad1* | 1539-2411 | 873 | 8 | 75.49 | -0.187 | 0.075 | ATA | TAA |
| *nad2* | 2495-3358 | 864 | 83 | 83.10 | -0.175 | 0.096 | ATA | TAA |
| *nad5* | 3410-4945 | 1536 | 51 | 80.92 | -0.199 | 0.003 | ATT | TAA |
| *nad6* | 4968-5420 | 453 | 22 | 83.89 | -0.153 | 0.315 | ATA | TAA |
| *nad3* | 5474-5794 | 321 | 53 | 79.44 | -0.176 | 0.061 | ATA | TAA |
| *cob* | 5812-6879 | 1068 | 17 | 75.56 | -0.180 | 0.027 | ATA | TAA |
| *cox3* | 6879-7670 | 792 | -1 | 76.89 | -0.225 | 0.060 | ATA | TAA |
| *atp6* | 7670-8311 | 642 | -1 | 80.37 | -0.233 | 0.032 | ATA | TAA |
| *NCRI-1* | 8312-8622 | 311 | 0 | 82.64 | 0.066 | -0.148 |  |  |
| *cox2* | 8623-9282 | 660 | 0 | 76.06 | -0.060 | 0.038 | ATA | TAA |
| *nad4* | 9298-10503 | 1206 | 15 | 81.76 | -0.203 | 0.000 | ATT | TAA |
| *Pcob-1* | 10505-10548 | 44 | 1 | 90.91 | -0.200 | -0.500 |  |  |
| *NCRI-2* | 10549-10767 | 219 | 0 | 71.69 | -0.134 | 0.097 |  |  |
| *Pcox2-1* | 10768-10891 | 124 | 0 | 78.23 | -0.010 | 0.185 |  |  |
| *NCRI-3* | 10892-11387 | 496 | 0 | 80.65 | -0.145 | 0.229 |  |  |
| *Pcox1-1* | 11388-11509 | 122 | 0 | 72.13 | 0.205 | -0.176 |  |  |

^a^ genes and pseudogenes located in the different strand from that of *cox1* are underlined. ^b^ inc = intergenic nucleotides, indicates gap nucleotides (positive value) or overlapped nucleotides (negative value) between two adjacent genes. ^c^ AT-skew = (A-T)/(A+T), GC-skew = (G-C)/(G+C).

B. Mitochondria chromosome II of *L. entomophila*

| gene^d^ | region | size | INC | AT% | AT-skew^b^ | GC-skew^c^ | start codon | stop codon |
| --- | --- | --- | --- | --- | --- | --- | --- | --- |
| *atp8* | 1-162 | 162 | 34 | 83.95 | -0.118 | -0.385 | ATA | TAA |
| *rrnL* | 163-1287 | 1125 | 0 | 80.53 | 0.035 | 0.178 |  |  |
| *rrnS* | 1288-2021 | 734 | 0 | 76.70 | 0.066 | 0.053 |  |  |
| *Pcox1-2* | 2048-2894 | 847 | 26 | 72.61 | 0.207 | -0.026 |  |  |
| *NCRII-1* | 2895-3105 | 211 | 0 | 74.88 | -0.114 | 0.057 |  |  |
| *Pcox2-2* | 3106-3234 | 129 | 0 | 72.87 | -0.128 | 0.371 |  |  |
| *NCRII-2* | 3235-4166 | 932 | 0 | 81.97 | -0.097 | 0.107 |  |  |
| *trnQ* | 4167-4218 | 52 | 0 | 84.62 | -0.227 | -0.250 |  |  |
| *NCRII-3* | 4219-4818 | 600 | 0 | 78.83 | -0.057 | 0.039 |  |  |
| *trnF* | 4819-4881 | 64 | 0 | 81.25 | 0.038 | 0.333 |  |  |
| *trnY* | 4937-4998 | 62 | 54 | 87.10 | 0.037 | 0.000 |  |  |
| *NCRII-4* | 4999-5267 | 269 | 0 | 82.16 | 0.131 | 0.042 |  |  |
| *Pcox1-3* | 5268-5458 | 191 | 0 | 69.11 | -0.303 | 0.017 |  |  |
| *Pnad5* | 5513-5644 | 132 | 54 | 77.27 | -0.255 | 0.333 |  |  |
| *NCRII-5* | 5645-5827 | 183 | 0 | 73.22 | 0.134 | -0.143 |  |  |
| *trnD* | 5828-5894 | 67 | 0 | 91.04 | 0.049 | 0.333 |  |  |
| *NCRII-6* | 5895-6028 | 134 | 0 | 82.09 | -0.036 | -0.167 |  |  |
| *trnS1* | 6029-6084 | 56 | 0 | 75.00 | -0.143 | -0.143 |  |  |
| *NCRII-7* | 6085-6580 | 496 | 0 | 77.42 | -0.099 | -0.036 |  |  |
| *Patp6-1* | 6581-6629 | 49 | 0 | 75.51 | -0.189 | 0.167 |  |  |
| *NCRII-8* | 6630-7109 | 480 | 0 | 76.04 | -0.052 | -0.009 |  |  |
| *Patp6-2* | 7110-7207 | 98 | 0 | 81.63 | 0.225 | -0.111 |  |  |
| *NCRII-9* | 7206-7659 | 452 | 0 | 79.20 | -0.039 | 0.085 |  |  |
| *trnL1* | 7660-7734 | 76 | 0 | 77.33 | -0.069 | 0.059 |  |  |
| *trnI* | 7706-7773 | 68 | -29 | 82.35 | 0.071 | -0.167 |  |  |
| *NCRII-10* | 7774-8003 | 230 | 0 | 79.13 | 0.022 | 0.042 |  |  |
| *trnT* | 8004-8060 | 57 | 0 | 89.47 | -0.059 | 0.333 |  |  |
| *Pcob-2* | 8110-8295 | 186 | 49 | 72.04 | 0.239 | -0.192 |  |  |
| *Pcob-3* | 8391-8597 | 207 | 95 | 81.16 | -0.095 | -0.128 |  |  |
| *NCRII-11* | 8598-8786 | 189 | 0 | 65.61 | 0.065 | -0.015 |  |  |
| *Pnad4* | 8787-8911 | 125 | 0 | 78.40 | 0.000 | 0.333 |  |  |
| *NCRII-12* | 8912-9267 | 356 | 0 | 81.46 | 0.062 | 0.000 |  |  |
| *Pcox3* | 9268-9341 | 74 | 0 | 81.08 | -0.233 | 0.000 |  |  |
| *NCRII-13* | 9342-10075 | 734 | 0 | 75.20 | 0.087 | -0.066 |  |  |
| *Pnad2* | 10076-10183 | 108 | 0 | 84.26 | 0.165 | -0.176 |  |  |
| *NCRII-14* | 10182-10315 | 132 | 0 | 81.06 | 0.140 | -0.120 |  |  |
| *Pcob-4* | 10316-10434 | 119 | 0 | 67.23 | 0.125 | -0.282 |  |  |
| *NCRII-15* | 10435-10643 | 209 | 0 | 85.65 | 0.128 | 0.267 |  |  |
| *trnM* | 10644-10710 | 67 | 0 | 71.64 | 0.000 | 0.053 |  |  |
| *NCRII-16* | 10711-11589 | 879 | 0 | 78.73 | 0.029 | 0.123 |  |  |
| *trnL2* | 11590-11653 | 64 | 0 | 82.81 | 0.019 | 0.091 |  |  |
| *NCRII-17* | 11654-12017 | 364 | 0 | 86.81 | -0.019 | -0.042 |  |  |
| *trnS2* | 12018-12070 | 53 | 0 | 90.57 | 0.042 | 0.200 |  |  |
| *trnK* | 12111-12169 | 59 | 40 | 79.66 | -0.277 | 0.167 |  |  |
| *NCRII-18* | 12169-12307 | 138 | 0 | 76.81 | -0.057 | -0.063 |  |  |
| *trnR* | 12308-12370 | 63 | 0 | 82.54 | -0.077 | 0.273 |  |  |
| *NCRII-19* | 12369-12576 | 206 | 0 | 79.13 | -0.117 | 0.070 |  |  |
| *trnP* | 12577-12641 | 65 | 0 | 81.54 | -0.132 | 0.333 |  |  |

^d^ genes and pseudogenes located in the different strand from that of *atp8* are underlined. ^b^ inc = intergenic nucleotides, indicates gap nucleotides (positive value) or overlapped nucleotides (negative value) between two adjacent genes. ^c^ AT-skew = (A-T)/(A+T), GC-skew = (G-C)/(G+C).
