# Supplementary material for: Evolution of multipartite mitochondrial genomes in the booklice of the genus Liposcelis (Psocoptera)
Source: BMC Genomics. 2014 Oct 5;15(1):861. doi: 10.1186/1471-2164-15-861 (PMC4197233; doi:10.1186/1471-2164-15-861)
Supplement: Supplementary file 8 — Additional file 8: Summary of the mitochondrial genome of Liposcelis paeta . agenes and pseudogenes located in the different strand from that of cox1 are underlined. binc = intergenic nucleotides, indicates gap nucleotides (positive value) or overlapped nucleotides (negative value) between two adjacent genes. cAT-skew = (A-T)/(A + T), GC-skew = (G-C)/(G + C). dgenes and pseudogenes located in the different strand from that of nad3 are underlined. (DOC 122 KB) [file 12864_2014_6535_MOESM8_ESM.doc]

Additional file 8. Mitochondrial genome of *Liposcelis paeta*

A Mitochondria chromosome I of *L. paeta*

| gene^a^ | region | size | INC | AT% | AT-skew^b^ | GC-skew^c^ | start codon | stop codon |
| --- | --- | --- | --- | --- | --- | --- | --- | --- |
| *cox3* | 1-795 | 795 | 0 | 72.05 | -0.059 | -0.010 | TTG | TAA |
| *cox1* | 795-2324 | 1530 | -1 | 67.06 | -0.228 | -0.004 | ATA | TAG |
| *NCRI-1* | 2325-2626 | 302 | 0 | 73.51 | 0.036 | 0.050 |  |  |
| *cob* | 2627-3679 | 1053 | 0 | 74.93 | -0.212 | -0.053 | ATA | TAA |
| *nad2* | 3707-4549 | 843 | 27 | 80.55 | -0.161 | 0.012 | ATA | TAA |
| *trnH* | 4574-4634 | 61 | 24 | 86.89 | -0.132 | 0.250 |  |  |
| *rrnL* | 4635-5659 | 1025 | 0 | 80.59 | 0.053 | 0.216 |  |  |
| *nad1* | 5660-6562 | 903 | 0 | 74.97 | -0.261 | 0.027 | ATT | TAA |
| *nad4* | 6562-7773 | 1212 | -1 | 76.57 | -0.203 | 0.028 | ATA | TAA |
| *trnS2* | 7772-7833 | 62 | -2 | 75.81 | 0.064 | 0.067 |  |  |
| *NCRI-2* | 7834-8065 | 232 | 0 | 71.55 | 0.012 | 0.394 |  |  |
| *cox2* | 8066-8752 | 687 | 0 | 74.47 | -0.247 | -0.094 | ATA | TAG |
| *atp8* | 8737-8895 | 159 | -16 | 77.36 | -0.220 | -0.222 | ATA | TAG |
| *atp6* | 8858-9514 | 657 | -38 | 75.34 | -0.228 | -0.025 | ATA | TAA |
| *nad5* | 9575-11080 | 1506 | 60 | 75.96 | -0.178 | -0.039 | ATT | TAA |
| *NCRI-3* | 11081-11211 | 131 | 0 | 70.23 | -0.130 | 0.026 |  |  |
| *PrrnL-1* | 11212-11399 | 188 | 0 | 75.00 | 0.092 | -0.277 |  |  |
| *trnQ* | 11437-11500 | 64 | 37 | 78.13 | -0.160 | 0.143 |  |  |

^a^ genes and pseudogenes located in the different strand from that of *cox3* are underlined. ^b^ inc = intergenic nucleotides, indicates gap nucleotides (positive value) or overlapped nucleotides (negative value) between two adjacent genes. ^c^ AT-skew = (A-T)/(A+T), GC-skew = (G-C)/(G+C).

B Mitochondria chromosome II of *L. paeta*

| gene^d^ | region | size | INC | AT% | AT-skew^b^ | GC-skew^c^ | start codon | stop codon |
| --- | --- | --- | --- | --- | --- | --- | --- | --- |
| *nad3* | 1-315 | 315 | 0 | 80.95 | -0.169 | -0.267 | ATA | TAA |
| *NCRII-1* | 316-573 | 258 | 0 | 68.99 | -0.034 | 0.050 |  |  |
| *trnT* | 574-647 | 74 | 0 | 82.43 | -0.148 | 0.385 |  |  |
| *trnV* | 689-757 | 69 | 41 | 81.16 | 0.000 | 0.231 |  |  |
| *NCRII-2* | 758-1147 | 390 | 0 | 70.00 | 0.011 | -0.179 |  |  |
| *trnK* | 1148-1203 | 56 | 0 | 80.36 | -0.200 | 0.455 |  |  |
| *NCRII-3* | 1204-1609 | 406 | 0 | 77.34 | -0.006 | 0.087 |  |  |
| *Patp6* | 1610-2171 | 562 | 0 | 74.91 | -0.230 | -0.007 |  |  |
| *NCRII-4* | 2172-2450 | 279 | 0 | 70.97 | 0.030 | -0.111 |  |  |
| *Pcox3* | 2451-2872 | 422 | 0 | 74.17 | 0.304 | 0.156 |  |  |
| *NCRII-5* | 2873-3866 | 994 | 0 | 73.64 | 0.074 | -0.015 |  |  |
| *trnL2* | 3867-3943 | 77 | 0 | 75.32 | -0.103 | 0.263 |  |  |
| *Pcob-1* | 4043-4104 | 62 | 99 | 70.97 | -0.364 | 0.000 |  |  |
| *NCRII-6* | 4105-4226 | 122 | 0 | 72.95 | 0.056 | 0.333 |  |  |
| *trnR* | 4227-4292 | 66 | 0 | 80.30 | -0.170 | 0.077 |  |  |
| *Pnad1* | 4297-4358 | 62 | 4 | 80.43 | -0.054 | -0.222 |  |  |
| *NCRII-7* | 4359-4609 | 251 | 0 | 69.72 | -0.154 | -0.026 |  |  |
| *Pcob-2* | 4610-4701 | 92 | 0 | 82.26 | -0.294 | -0.818 |  |  |
| *trnY* | 4768-4825 | 58 | 66 | 81.03 | -0.064 | -0.091 |  |  |
| *NCRII-8* | 4826-5217 | 392 | 0 | 75.26 | -0.166 | 0.010 |  |  |
| *trnC* | 5218-5277 | 60 | 0 | 86.67 | 0.000 | 0.250 |  |  |
| *NCRII-9* | 5278-6258 | 981 | 0 | 72.78 | -0.092 | -0.049 |  |  |
| *trnF* | 6259-6317 | 59 | 0 | 89.83 | 0.208 | 0.333 |  |  |
| *PrrnL-2* | 6368-7287 | 914 | 50 | 80.09 | 0.041 | 0.198 |  |  |
| *trnG* | 7380-7446 | 67 | 92 | 88.06 | -0.017 | 0.000 |  |  |
| *nad4L* | 7447-7693 | 247 | 0 | 81.38 | -0.154 | 0.217 | ATA | T |
| *trnM* | 7692-7757 | 66 | -2 | 78.79 | 0.038 | 0.000 |  |  |
| *rrnS* | 7758-8462 | 705 | 0 | 78.87 | 0.004 | -0.154 |  |  |
| *trnA* | 8463-8524 | 62 | 0 | 83.87 | 0.192 | 0.200 |  |  |
| *nad6* | 8622-9101 | 480 | 97 | 81.88 | -0.262 | 0.103 | ATA | TAA |
| *Pnad3* | 9103-9266 | 164 | 1 | 83.54 | 0.212 | 0.111 |  |  |
| *NCRII-10* | 9267-10322 | 1056 | 0 | 76.23 | 0.026 | -0.068 |  |  |

^d^ genes and pseudogenes located in the different strand from that of *nad3* are underlined. ^b^ inc = intergenic nucleotides, indicates gap nucleotides (positive value) or overlapped nucleotides (negative value) between two adjacent genes. ^c^ AT-skew = (A-T)/(A+T), GC-skew = (G-C)/(G+C).
