# Supplementary figures and images for: Evolution of multipartite mitochondrial genomes in the booklice of the genus Liposcelis (Psocoptera)
Source: BMC Genomics. 2014 Oct 5;15(1):861. doi: 10.1186/1471-2164-15-861 (PMC4197233; doi:10.1186/1471-2164-15-861)

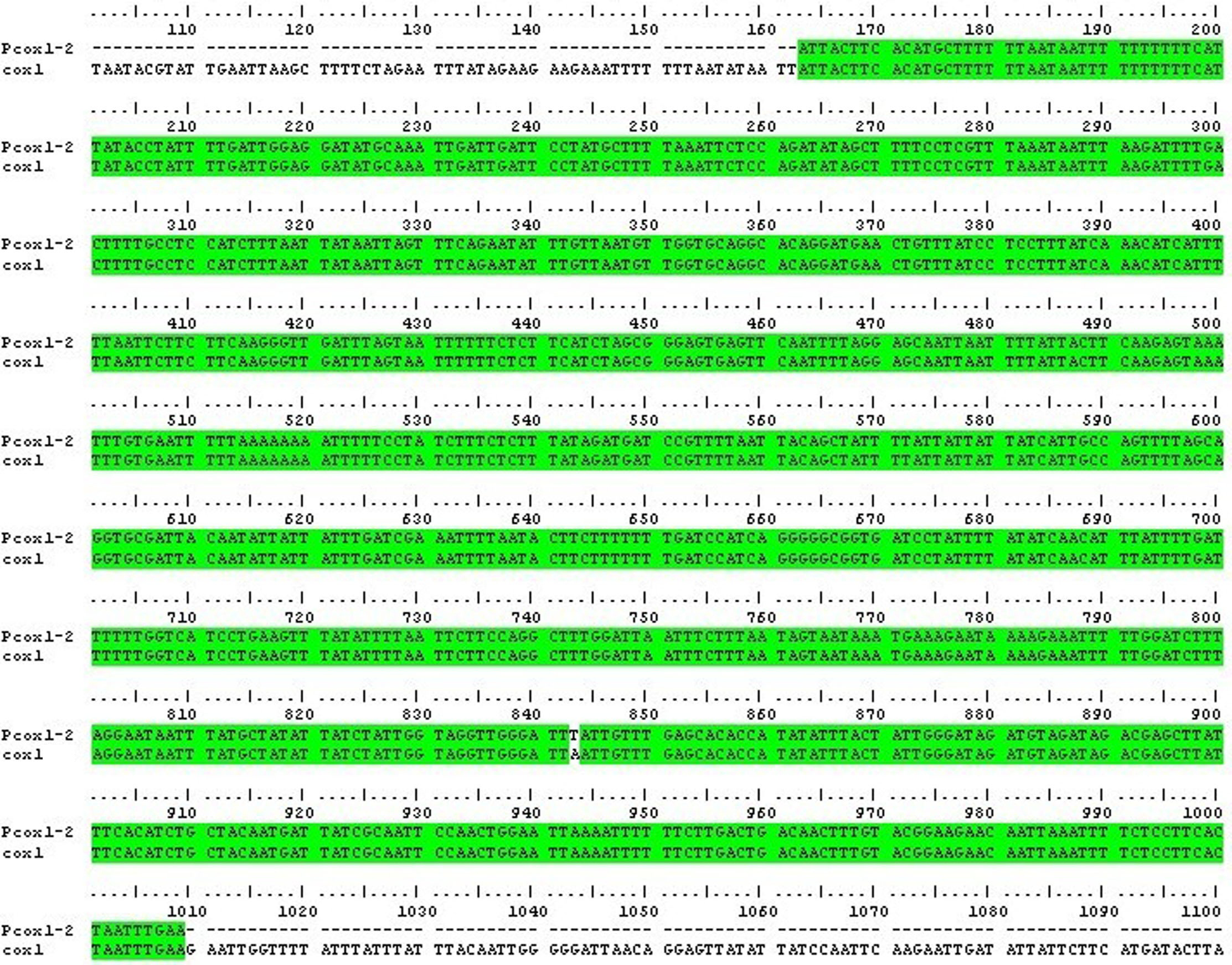

Supplement: Supplementary file 9 — Additional file 9: Alignments of putative the pseudogene Pcox1-2 and putative functional gene cox1 of Liposcelis entomophila . Consensus sequences are shown in the green background. (TIFF 5 MB) [file 12864_2014_6535_MOESM9_ESM.tiff]
